# First Episode Psychosis and Pituitary Hyperplasia in a Patient With Untreated Hashimoto’s Thyroiditis: A Case Report

**DOI:** 10.3389/fpsyt.2022.863898

**Published:** 2022-03-24

**Authors:** Celeste Lipkes, Shanzay Haider, Ali Rashid, Gustavo A. Angarita, Sarah Riley

**Affiliations:** ^1^Department of Psychiatry, Yale University School of Medicine, New Haven, CT, United States; ^2^Department of Mental Health, Charles George Veterans Affairs Medical Center, Asheville, NC, United States; ^3^Department of Endocrinology, Yale University School of Medicine, New Haven, CT, United States; ^4^Clinical Neuroscience Research Unit, Connecticut Mental Health Center, New Haven CT, United States

**Keywords:** first episode psychosis, hypothyroidism, Hashimoto’s thyroiditis, myxedema madness, pituitary hyperplasia

## Abstract

This case report describes a woman with no psychiatric history and previously diagnosed Hashimoto’s thyroiditis who presented to the psychiatric emergency department with a first episode of psychosis. The initial workup for organic causes of psychosis revealed an astronomically high thyroid stimulating hormone (TSH) (> 1,000 μIU/mL) out of proportion to the patient’s minimal physical symptoms of hypothyroidism. Additionally the patient’s head imaging showed an enlarged pituitary, a rare, but reversible, presentation of chronically untreated primary hypothyroidism. The patient was transferred to a medical unit to receive IV thyroid hormone replacement as well as an adjunctive antipsychotic to assist with remission of her distressing auditory hallucinations and persecutory delusions. This case highlights the importance of a thorough medical workup for causes of new onset psychosis and the need for further consensus in the literature regarding choice of antipsychotic and duration of treatment for psychosis secondary to hypothyroidism.

## Highlights

– All patients with new onset psychosis should receive thyroid screening.
– Pituitary hyperplasia is a rare and reversible presentation of severe hypothyroidism.
– Treating patients with psychiatric symptoms secondary to thyroid illness will benefit from a multi-specialty approach, including teams from psychiatry, endocrinology, and in rare cases ophthalmology.
– Many patients with psychotic symptoms secondary to hypothyroid illness will benefit from taking short-term antipsychotics, however, there is no clear consensus on the preferred agent or length of treatment.

## Introduction

Alterations in the levels of thyroid hormones thyroxine (T4) and triiodothyronine (T3) can lead to multi-system effects, including neuropsychiatric disturbances such as psychosis. Though the underlying mechanism is not completely clear, both T4 and T3 are critical to maintaining adequate neuronal conduction and cerebral blood flow (1, 2) and low T4 or T3 have been associated with reduced glucose uptake in areas of the brain such as the amygdala and hippocampus (2). Psychosis is present in 5–15% of hypothyroid patients and was first described as early as 1888 by the Committee of the Clinical Society of London (3). In 1949, Dr. Richard Asher coined the term “myxedema madness” to describe psychosis secondary to hypothyroidism and described a wide range of mental changes that could accompany it (4). Psychotic symptoms in hypothyroid patients typically follow months to years of physical symptoms (5) and can include visual and auditory hallucinations, paranoia, (6) delusions such as Capgras syndrome (believing that a close family member has been replaced by an identical-looking imposter), (7) and disordered thought process, including perseveration and loose associations. A recent systematic review of case studies identified the median age of presentation of myxedema psychosis as 42 years, with the most common presenting feature being the presence of delusions (91%), the majority (84%) of which were paranoid or persecutory in nature. Approximately half of these patients did not have a hypothyroidism diagnosis at presentation, and 37% of patients presented without physical symptoms. The maximum thyroid stimulating hormone (TSH) level identified was 139 μIU/mL, with a median of 93. Notably, 89% of patients had normal head imaging (8).

## Case Description

Our patient is a 29-year-old female with hypothyroidism and no psychiatric history who was sent to the Emergency Department by Urgent Care for one week of new-onset psychotic symptoms.

Upon initial presentation to the urgent care clinic, the patient endorsed auditory hallucinations (“I’m hearing other people’s conversations and they are not saying anything to comfort me!”). Her vital signs and physical examination were normal. She was diagnosed with “acute paranoia” and sent to Yale New Haven Hospital for evaluation, where she was directly triaged to the psychiatric emergency department.

Upon evaluation by a psychiatrist, the patient endorsed a one-week history of hearing people speaking negatively about her and resultant worsening anxiety. She denied command auditory hallucinations, suicidality, and homicidality. She denied any mood symptoms or changes in sleep or appetite. She made several odd statements about being followed that she was unable to explain. She endorsed sporadic THC use, last used over two months ago, and denied all other substance use. She denied any prior personal or familial history of psychosis. She had fair insight into her family’s concern and gave consent for the team to contact collateral sources of information. Her boyfriend and her sister reported that for the prior few days the patient had skipped work because she believed people were talking about her. They also noted that the patient seemed suspicious and would ask them repeatedly, “did you hear that?” in an otherwise silent room. On physical exam, her cranial nerves were intact with normal testing of her visual fields. She endorsed mild fatigue and cold intolerance and denied other symptoms of hypothyroidism. On mental status exam, she was pleasant, anxious-appearing, paranoid, and fully oriented.

## Diagnostic Assessment and Intervention

Initial workup for our patient included a urine toxicology screen (including negative THC), urine pregnancy test, and COVID swab, which were unremarkable. Prior to the patient’s CBC, BMP, TSH, and T4 resulting, a head CT was completed showing “enlargement of the pituitary gland (measuring 1.3 cm craniocaudally) with suprasellar component encroaching upon the optic chiasm.” Neurosurgery was consulted and recommended an MRI brain with pituitary protocol as well as endocrine and ophthalmology consults. Meanwhile, the patient became increasingly paranoid, anxious, and tearful, stating she had “revealed too much, and her life was ruined.” The initial laboratory results then returned significant for a normal morning cortisol, prolactin of 114 ng/mL, TSH of >1,000 μIU/mL, and a low free thyroxine (FT4) of 0.25 ng/dL. Upon further questioning, the patient confirmed a history of Hashimoto’s thyroiditis diagnosed at age 18. Pre-electronic medical records corroborated a history of elevated thyroid peroxidase antibodies, confirming this diagnosis.

**FIGURE 1:**
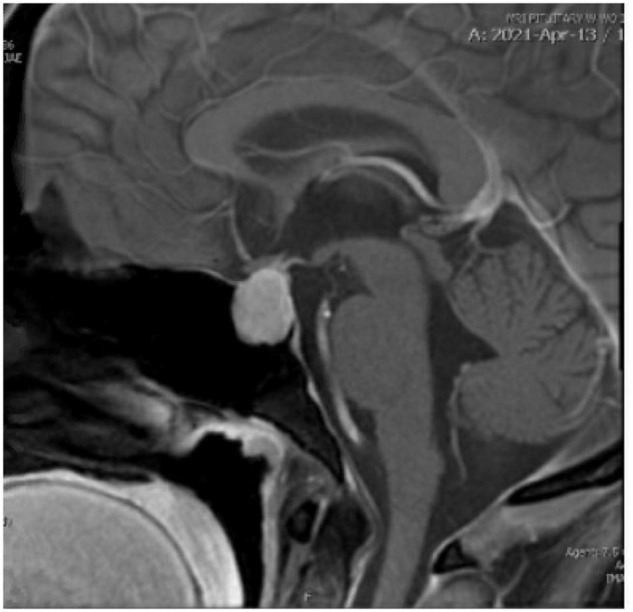
The patient’s initial MRI with pituitary protocol.

On examination by the endocrine team, her extraordinarily elevated TSH was out of proportion to both her reported hypothyroid symptoms and physical exam, which revealed only mild puffiness of the face, pale color, and a delayed relaxation phase in her deep tendon reflexes. The MRI with pituitary protocol returned, showing “a 2.3 cm enhancing intrasellar mass with extension into bilateral cavernous sinuses and suprasellar compartment contacting the optic chiasm, likely to represent a pituitary macroadenoma” (see Figure 1). The endocrine team determined the enlargement of the patient’s pituitary seen on imaging was physiologic in the setting of primary hypothyroidism and neurosurgical intervention was not required. Chart review and discussion with the patient revealed she was diagnosed with Hashimoto’s thyroiditis in 2017 and self-discontinued her levothyroxine a year later without follow-up. The patient stated that prior to arrival to the emergency department she had taken several of her levothyroxine pills to see if it would make her feel better. The endocrinology team felt her profound hypothyroidism was the cause of her new onset psychotic symptoms, and she was medically admitted for IV levothyroxine treatment.

During the patient’s eight-day admission, she was followed by endocrinology, ophthalmology, and psychiatry consult services. She received three days of 100mcg IV levothyroxine before transitioning to her home dose of 150mcg levothyroxine daily. The ophthalmology team completed formal testing of the patient’s visual fields, which were normal. On evaluation by psychiatry, the patient initially endorsed a decrease in her auditory hallucinations but reported ongoing paranoid thoughts (doctors were listening to her on the phone, her food had been laced with laxatives in the emergency department, etc.). Despite these delusions about her care team, the patient was amenable to workup and treatment by all her providers, including our team’s recommendation that she trial an antipsychotic. The patient took aripiprazole, titrated to 5 mg, for two days without significant improvement in her paranoia. She was then switched to risperidone 1 mg BID; after two days on this regimen, she denied all paranoid thoughts. When asked if people were speaking ill of her or trying to poison her food, she stated “I have no idea how I could have thought that.” She showed good insight into her prior delusions and the need for ongoing hypothyroid treatment. She was referred to outpatient psychiatric care and was scheduled for endocrine labs and follow-up post discharge.

One month after discharge, the patient was found to have normal TSH (4.070 μIU/L). Given an elevated FT4 (1.76 ng/dL), her levothyroxine was reduced to 125 mcg daily.

Two months after discharge, she followed up in the endocrinology clinic and reported compliance to both levothyroxine and risperidone. She endorsed hearing only rare voices, though felt this was because she was “overthinking things” rather than experiencing true auditory hallucinations. She denied paranoia and had returned to work full time.

The patient was lost to formal psychiatric follow-up; however four months after discharge, she was contacted by a member of our team, which she had previously consented to. At this time, she endorsed stopping risperidone about two months after discharge, around when her auditory hallucinations completely resolved. She denied any symptoms of psychosis, was compliant with daily thyroid replacement, and was pleased to relay the results of her follow-up MRI: her pituitary measured 7mm in height and the hyperplasia had fully resolved.

## Discussion

### Thyroid Hormone and Neuropsychological Conditions

Our patient’s presentation was suspicious for an organic psychosis, given her lack of prodromal symptoms, no history of familial psychotic illness, and history of thyroid dysfunction. A thyroid panel should be included in every standard workup for new-onset psychosis; our patient’s initial labs revealed an astronomically high TSH—the highest ever seen by Yale endocrinology and significantly higher than levels seen in similar case reports. When a TSH is noted to be elevated, assessment of T4 levels is the next step in assessing the hypothalamic-pituitary-thyroid axis. In our patient, her T4 was 0.25 ng/dL, which was likely detectable only because of the levothyroxine the patient had taken before arrival to the hospital.

The sequelae of hypothyroidism exist on a spectrum ranging from minimal symptoms to florid myxedema coma. It is important to recognize myxedema coma early, as mortality rates can be as high as 50% (9). The magnitude in altered TSH and T4 levels are poorly correlated with severity of symptoms. Notably, the diagnosis of myxedema encephalopathy, or coma, is based solely on clinical evaluation. Clinical features which raise concern for myxedema and warrant a higher level of care include altered mental status, presence of hypothermia, and preceding events like infection, which can unmask underlying severe hypothyroidism (9). A scoring system for myxedema is present in the literature and can be useful in aiding whether myxedema coma is likely (10). However, this scoring system has not been repeatedly validated within the literature.

Thyroid hormone has several roles within the central and peripheral nervous systems, which can explain the varying degrees of neurological symptoms associated with hypothyroidism, such as depression, delayed reflexes, etc. (1). There is speculation about whether antibodies present in autoimmune thyroid disease, specifically Hashimoto’s thyroiditis, may have a role in altered neurological function independent of thyroid hormone levels (11). However, current literature is conflicting and has not shown a clear causative effect. Irrespective of the cause, long-standing untreated hypothyroidism seems to be most consistently associated with neurological symptoms.

### Hypothyroidism and Pituitary Hyperplasia

Another unique feature of this case is the presence of pituitary hyperplasia on imaging, which is a known but rare presentation of chronically untreated primary hypothyroidism. It is typically seen in patients with Hashimoto’s disease when there is underproduction of thyroid hormone from the thyroid gland. This deficiency signals the hypothalamus to produce thyrotropin releasing hormone (TRH) to stimulate the thyrotropes within the pituitary gland to produce TSH. Normally TSH would then bind to its receptor on the thyroid gland and produce both T4 and T3, which suppresses further release of TRH. However in primary hypothyroidism, the thyroid gland does not produce thyroid hormone in response to TSH. In chronic primary hypothyroidism, there is a loss of T4-mediated suppression of TRH which leads to tonic stimulation of the pituitary gland and subsequent hypertrophy. Of note, TRH also stimulates the lactotrophe cells within the pituitary gland which produce prolactin (PRL). As noted in this case, patients can often have a mild elevation in PRL. With appropriate treatment, there should be normalization of both TSH and PRL as well as resolution of the pituitary hypertrophy—as seen in our patient in follow up imaging.

### Treatment of Hypothyroidism and Controversies in Psychosis Management

Under the guidance of the endocrinology team, the patient was initially treated with intravenous (IV) levothyroxine and transitioned to a weight-based dose of oral levothyroxine on discharge. The use of IV levothyroxine is typically reserved for treatment of severe hypothyroidism or myxedema coma. However, IV levothyroxine also has a role in individuals who cannot tolerate oral levothyroxine for prolonged periods of time while hospitalized. Once clinically improved, patients can be transitioned to oral levothyroxine. It is typically recommended to start an oral dose of 1.6 mcg/kg in an otherwise healthy, young individual. Clinicians should consider a reduced dose of levothyroxine in those patients who are elderly or have underlying cardiac conditions, as levothyroxine can be arrhythmogenic (1). Other therapies such a liothyronine and steroids are typically reserved for those who are critically ill requiring step-down or intensive care. Therapies such as myoinositol and selenium are not typically prescribed in the acute setting and are not standard of care by endocrine society guidelines. Thyroidectomy is not recommended in acute treatment of hypothyroidism, as it has little clinical impact on disease.

While intravenous levothyroxine was clearly indicated for the patient’s severe hypothyroidism, it was less obvious how best to manage her psychotic sequelae. A majority of similar case reports of patients with delusions and hallucinations secondary to hypothyroidism describe the use of short-term antipsychotics to minimize psychotic symptoms. Antipsychotic augmentation of thyroid replacement may be especially helpful in elderly patients—whose neuropsychiatric symptoms often take longer to resolve and can even become permanent—and patients like ours whose psychotic symptoms are distressing, disabling, or likely would interfere with their ability to comply with a daily thyroid medication (12).

There is no clear standard of care in the literature for preferred antipsychotic agent or length of treatment; in several reports both low dose typical (haloperidol) and atypical (risperidone, olanzapine) antipsychotic medications were administered over a period of weeks. Initially, the team selected low dose aripiprazole for our patient due to concern over exacerbating her elevated prolactin (114). Some guidelines suggest that when antipsychotic-induced elevated prolactin levels are >50 or there are clinical symptoms, medication such as aripiprazole should be added to reduce prolactin levels; although this case was not antipsychotic-induced, initial agent selection was based upon this principle (13). After multiple days receiving low dose aripiprazole without reduction in her psychotic symptoms, she was switched to risperidone, given several case studies suggesting clinical improvement within two weeks’ duration on this regimen (12). So long as a patient is not experiencing symptoms such as infertility from elevated prolactin, medications with a higher risk of prolactin elevation can be safely used. However, upon review, given the patient’s female gender, pituitary hyperplasia, and prolactin levels <100, lengthier trial of aripiprazole instead of switching to risperidone may have been warranted. Guidance on initial antipsychotic selection and criteria for switching in psychotic patients with hyperprolactinemia secondary to hypothyroidism are needed, as highlighted by this case.

It was clear that after several days taking risperidone, the patient’s positive symptoms, particularly her paranoia, had significantly improved. Unfortunately, because the patient was lost to formal psychiatric follow-up, it is unclear exactly when her psychotic symptoms fully resolved, though she estimated that her auditory hallucinations completely remitted two months after her initial presentation, at which time she self-discontinued her risperidone.

## Data Availability Statement

The original contributions presented in the study are included in the article/supplementary material, further inquiries can be directed to the corresponding author.

## Ethics Statement

Written informed consent was obtained from the individual(s) for the publication of any potentially identifiable images or data included in this article.

## Author Contributions

CL, SR, GA, and AR contributed to initial psychiatric care and workup of the patient in the Crisis Intervention Unit, conceptualization of the case report, and writing and editing it. SH contributed to the medical care of the patient, conceptualization of the case report, and writing and editing it. All authors contributed to the article and approved the submitted version.

## Conflict of Interest

The authors declare that the research was conducted in the absence of any commercial or financial relationships that could be construed as a potential conflict of interest.

## Publisher’s Note

All claims expressed in this article are solely those of the authors and do not necessarily represent those of their affiliated organizations, or those of the publisher, the editors and the reviewers. Any product that may be evaluated in this article, or claim that may be made by its manufacturer, is not guaranteed or endorsed by the publisher.

## References

[1] Melmed SA Goldfine A Koenic R Rosen C. *Williams Textbook of Endocrinology.* 14th ed. Philadelphia, PA: Elsevier (2020). p. 404–32.

[2] Bauer M Silverman DH Schlagenhauf F London ED Geist CL van Herle K Brain glucose metabolism in hypothyroidism: a positron emission tomography study before and after thyroid hormone replacement therapy. *J Clin Endocrinol Metab.* (2009) 94:2922–9. 10.1210/jc.2008-2235 19435829

[3] Humphry G. Report of a Committee of the Clinical Society of London. *J Anatomy Physiol.* (1886) 20(Pt 3):546.

[4] Asher R. Myxoedematous madness. *Br Med J.* (1949) 2:555. 18148089 10.1136/bmj.2.4627.555 PMC2051123

[5] Azzopardi L Murfin C Sharda A De Silva N. Myxoedema madness. *BMJ Case Rep.* (2010) 2010:bcr0320102841. 10.1136/bcr.03.2010.2841 22778250 PMC3028477

[6] Heinrich TW Grahm G. Hypothyroidism presenting as psychosis: myxedema madness revisited. *Prim Care Companion J Clin Psychiatry.* (2003) 5:260–6. 10.4088/pcc.v05n0603 15213796 PMC419396

[7] Madakasira S Hall TB III. Capgras syndrome in a patient with myxedema. *Am J Psychiatry.* (1981) 138:1506–8. 7294225 10.1176/ajp.138.11.1506

[8] Mohamed MFH Danjuma M Mohammed M Mohamed S Siepmann M Barlinn K Myxedema psychosis: systematic review and pooled analysis. *Neuropsychiatr Dis Treat.* (2021) 17:2713–28. 34447249 10.2147/NDT.S318651 PMC8382967

[9] Ono Y Ono S Yasunaga H Matsui H Fushimi K Tanaka Y. Clinical characteristics and outcomes of myxedema coma: analysis of a national inpatient database in Japan. *J Epidemiol.* (2017) 27:117–22. 10.1016/j.je.2016.04.002 28142035 PMC5350620

[10] Popoveniuc G Chandra T Sud A Sharma M Blackman MR Burman KD A diagnostic scoring system for myxedema coma. *Endocr Pract.* (2014) 20:808–17. 24518183 10.4158/EP13460.OR

[11] Churilov LP Sobolevskaia PA Stroev YI. Thyroid gland and brain: Enigma of Hashimoto’s encephalopathy. *Best Pract Res Clin endocrinol Metab.* (2019) 33:101364. 10.1016/j.beem.2019.101364 31801687

[12] Hynicka LM. Myxedema madness: a case for short-term antipsychotics? *Ann Pharmacother.* (2015) 49:607–8. 10.1177/1060028015570089 25870443

[13] Dehelean L Romosan A-M Papava I Bredicean CA Dumitrascu V Ursoniu S Prolactin response to antipsychotics: an inpatient study. *PLoS One.* (2020) 15:e0228648. 10.1371/journal.pone.0228648 32017792 PMC6999917

